# Supplementary material for: Nutritional and physicochemical characteristics of purple sweet corn juice before and after boiling
Source: PLoS One. 2020 May 11;15(5):e0233094. doi: 10.1371/journal.pone.0233094 (PMC7213738; doi:10.1371/journal.pone.0233094)
Supplement: S1 Table — (DOCX) [file pone.0233094.s001.docx]

**S1 Table. Two-way anova of free phenols, anthocyanins and antioxidant activity.**

| **Variate: Phenols** | |  | |  | |  |  |  |
| --- | --- | --- | --- | --- | --- | --- | --- | --- |
| Source of variation | SS | df | | MS | | F | P-value | F crit |
| Raw/Boiled | 1.69E-02 | 1 | | 1.69E-02 | | 134.462 | 0.000 | 5.318 |
| Frozen/Fresh | 2.82E-03 | 1 | | 2.82E-03 | | 22.481 | 0.001 | 5.318 |
| interactive | 1.05E-03 | 1 | | 1.05E-03 | | 8.329 | 0.020 | 5.318 |
| Residual | 1.00E-03 | 8 | | 1.26E-04 | |  |  |  |
| Total | 2.17E-02 | 11 | |  | |  |  |  |
| **Variate: Anthocyanins** | | | |  | |  |  |  |
| Source of variation | SS | df | | MS | | F | P-value | F crit |
| Raw/Boiled | 5.33E-06 | 1 | | 5.33E-06 | | 0.137 | 0.721 | 5.318 |
| Frozen/Fresh | 4.81E-04 | 1 | | 4.81E-04 | | 12.342 | 0.008 | 5.318 |
| interactive | 4.08E-04 | 1 | | 4.08E-04 | | 10.470 | 0.012 | 5.318 |
| Residual | 3.12E-04 | 8 | | 3.90E-05 | |  |  |  |
| Total | 1.21E-03 | 11 | |  | |  |  |  |
| **Variate: Antioxidant-DPPH** | | | |  | |  |  |  |
| Source of variation | SS | | df | MS | | F | P-value | F crit |
| Raw/Boiled | 563.139 | | 1 | 563.139 | | 158.621 | 0.000 | 5.318 |
| Frozen/Fresh | 37.386 | | 1 | 37.386 | | 10.531 | 0.012 | 5.318 |
| interactive | 8.255 | | 1 | 8.255 | | 2.325 | 0.166 | 5.318 |
| Residual | 28.402 | | 8 | 3.550 | |  |  |  |
| Total | 637.182 | | 11 |  | |  |  |  |
| **Variate: Antioxidant-Trihydroxybenzene** | | | | | | |  |  |
| Source of variation | SS | df | | MS | F | | P-value | F crit |
| Raw/Boiled | 106.273 | 1 | | 106.273 | 137.015 | | 0.000 | 5.318 |
| Frozen/Fresh | 18.128 | 1 | | 18.128 | 23.372 | | 0.001 | 5.318 |
| interactive | 3.248 | 1 | | 3.248 | 4.187 | | 0.075 | 5.318 |
| Residual | 6.205 | 8 | | 0.776 |  | |  |  |
| Total | 133.854 | 11 | |  |  | |  |  |
