# Supplementary material for: Nutritional and physicochemical characteristics of purple sweet corn juice before and after boiling
Source: PLoS One. 2020 May 11;15(5):e0233094. doi: 10.1371/journal.pone.0233094 (PMC7213738; doi:10.1371/journal.pone.0233094)
Supplement: Attachment — Submitted filename: Comments.docx [file pone.0233094.s002.docx]

L24-44: The abstract should not include methodological details. Please rephrase/remove.
L25-28: Text: In order to contribute to the increased knowledge available for consumers on sweet corn juice, the nutritional and physicochemical characteristics of raw and boiled purple sweet juice was herein investigated.
L33-34: Refrigerated samples? Is this the freezed or raw samples? Both of those samples reach similar levels on anthocyanin after boiling. Please rephrase.
L40: Text: The boiling process had no … However, the content of fatty acids were …
L42-44: Text: These results provide detailed information relevant for further development of sweet corn juice …
L77: Text: Boiling and roasting has been found to…
L92: Is heat-blanching investigated in the present work? If not remove/rephrase.
L100-109: CAS numbers for the products and their purity/quality is missing.
L117: What type of juice extractor? Can I expect the same homogenization if I use my extractor at 10 000 rpm for 1 min?
L120: Medical gauze? Details? Pore-size? Material? This will affect the degree of filtration.
L126-129: Level of significance used (alpha value(s))? As mentioned in the guidelines, this should be specified.
L132: How is degradation and racemization of amino-acids accounted for in the applied method? Such effects will affect the estimated nutritional values of the juice.
L132-134: 1 mL of water was added to the dried/evaporated 1 mL aliquot, then added 1 mL of water before a second evaporation to dryness? This should be written clearer.
L134: What is the hydrolysate? The original liquid from which 1 mL of supernatant was withdrawn? Or the aliquot eventually evaporated? I guess both are hydrolysates and the difference/similarity should be stated clearer.
L140: What type of filter paper? Technical details?
L154: Equations should be numbered, referred to in the text, and described in detail.
L164: Ether-petroleum ether mixture? Diethyl ether mixed with petroleum ether? Which ratio?
L171: Added to the extraction and staying at 85 °C? Added to the separated and dried organic phase and kept at 85 °C for 30 min? Please clarify.
L172: Text: … and kept at 85 …
L177: Membrane material?
L181: Text: Heating to 200 °C…
L194: Text: Heating to …
L196: Text: Split ratio = unsplit; inlet temperature = 250 °C; ….
L219: Text: … to remove the acid.
L224: Text: Detector = DAD-FLD; column temperature = …
L239-240: Please give a short summary of the applied method.
L243: Technical details for medical gauze is missing.
L244: What are the units for the obtained viscosity?
L247: Why was absorbance used and why 660 nm? Which component in the juice absorbs light at this wavelength and why is this related to the stability of the juice?
L271: Table 1: Please clarify (with details, or correct) how there is a statistical difference (significance level of 0.05) between for example Raw vs. Boiled samples and their Methionine content considering the concentration in the juice is given as 297.62 ± 25.64 g/L vs. 298.96 ± 15.80 g/L, respectively. The same holds for Thr, Lys, Phe, Leu, …
L280: Text: Casein, a dairy protein…

L596: Figure 1 contain abbreviations not described in the text (VB1, VB2 and VE) and Figure text is missing for both Figure 1 and 2. Caption for Figure 2 is on L447, while caption for Figure 1 is missing. Please follow instructions given in the guidelines.
L301-303: I guess the values obtained for sweet and normal field corns are obtained from reference [5]? Anyways, please include the reference subsequent the states values, or at the end of L303.
L308: Text: … loss of vitamin B1 upon boiling and steaming…
L310: Text: … and only slightly affected by thermal processing.
L311: Text: … is easily degraded by light…
L312: Text: … is susceptible for chemical modifications in …
L321: Text: … in the germ, with a fraction of... being as high as…
L315: Text: … 35 different fatty acids were identified (fatty acids with a content less than 0.5 mg/L are not included)…
L330-333: Text: Considering the presence of aromatic compounds (Table 3), it was initially speculated that boiling enhanced fatty acid volatility, promoting their evaporation. This because …
L335-337: Please avoid personal pronouns. Text: However, it is believed that… What is the volatility of long chained fatty acids? Boiling points or vapour pressure for pure components? Text: Thus, the residues from … were also analysed.
L341: Avoid ‘we’.
L341-344: Very interesting!
L345: Text: On a dry weight basis, …
L358: Three biological replicates of what? Of raw and boiled purple sweet corn juice? Clarify.
L364: Text: The presence of most compounds varied…
L366-367: Text: …, suggesting they occurred in trace amounts.
L369: Text: … in the purple sweet …
L375: Table 3: Units are missing. Which internal standard was used during GC-MS for fatty acids and volatile components? Please include this information in the appropriate sections within the Materials and methods.
L382: Text: … which has been reported to occur persistently [References].
L384: Text: … whereas in the present work it was found to be a major component (~10%) within the aromatic compounds.
L386: References relating to the statement regarding aromatic compound composition is missing. Text: … for the purple sweet corn investigated in the present work and those of previously reported corns, …
L388-389: Text: …, thus enhancing properties related to desirable smells. In addition, boiling changes the composition of the volatile compounds through …
L393: In the raw corn juice, 54 compounds were exclusively identified, such as …
L395-396: Did you measure the overall aroma from the juices? If not I would change the text: Thus, a difference of aroma due to the boiling of purple sweet corn juice can be expected.
L399: Text: … (Table 4; Frozen juice)…
L402: Text: In the cited work, severe heat …
L404: Text: … lower temperature (95 °C for 5 min) was applied in the present work. The investigated process conditions could have been insufficient for the release of …
L405-406: Text: The change in anthocyanin content was also in contrast to what is reported in literature, as in general, anthocyanin’s are …
L407-409: Text: However, the present work found that boiling for 5 min increased the total anthocyanin content in purple sweet corn juice (Table 4; Frozen juice).
L415: Table 4: Regarding the anthocyanin content in frozen vs. fresh juice – what are possible explanations for the contradicting results apparently occurring due to the freezing process? How was the samples thawed after freezing (specify in Materials and methods)? The content of the boiled samples seem consistent (thus not being affected by the freezing) while the raw samples seem more ‘sensitive’ to the freezing process. Interesting!
L418-423: Even though enzymatic degradation may occur, thus explain any decrease of anthocyanin’s, the results suggest that freezing (which can be expected to correlate to low enzymatic activities) decrease the anthocyanin content more than that of raw samples. How long, and at which temperatures where the ‘raw’ and ‘frozen’ samples stored for/at? This should be specified in the Materials and Methods.
L426: Text: … contents decreased by …
L428: Text: … fresh juice decreased by …
L430: Text: … phenol content decreased by …
L431: Text: … content increased with …
L431: And consequently antioxidant capacity was decreased by 35.7 and 17.3% after boiling? Is antioxidant activity related the anthocyanin content? It was previously mentioned that the antioxidant content is related to the content of phenols. Please rewrite or provide references or data linking antioxidant activity with anthocyanin content, or compare the chemistry of phenol’s and anthocyanin’s.
L432-433: … after boiling as determined by the DPPH and …
L433: The improvement of antioxidant capacity of fresh raw juice as compared to what? The samples frozen and/or boiled? Which antioxidant capacity? Table 5 states that (P value of 0.012) the freezing not significantly affects (assuming a limit of significance equal to 0.05) the DPPH radical scavenging activity of the samples. I would rewrite L433 to something like: “The high antioxidant capacity observed for fresh raw juice samples (Table 4) seem to correlate with the elevated contents of phenols and anthocyanins”. However, the fact that frozen samples present phenol/anthocyanin content developments where their respective contents either decrease or increase due to the boiling treatment, while for raw samples both contents respond on boiling with a decrease does not justify the degradation statement on L434-436. Please rewrite.
L433: Text: … boiled juices are presented in Fig 2…
L444-445: Text: These results are consistent with previous…
L446: Regarding references: Please be consistent with the format used for the references.
L447: Place figure texts in accordance with author guidelines.
L448: Text: Asterix (*) indicates significant difference between raw and boiled samples…
L451: Please provide reference regarding the statement for gelatinization unless this was confirmed directly by any of the results in the present work.
L456-457: Rewrite the final sentence. Why is it desirable with elevated viscosity and stability? Is it beneficial that the appearance of beverages is homogenous? What about mouthfeel?
L459: Text: This study offer a comprehensive understanding on the…
L461: Was starch gelatinization measured directly? If not rewrite.
L461: Rewrite the statement on fragrance and aromatic compounds. The volatile components identified in the present work is given with a relative content, also, boiling only potentially enhance fragrance related properties as this not has been measured directly. Thus, a concluding sentence on the changes of volatile/aromatic potentially favouring enhanced smell-properties would seem more accurate.
L467-470: Was enzymatic activity in the raw material and the response to heat-blanching tested in the present work? If not remove this from the conclusion (could be moved to discussion).
L471-474: This can be moved to the part covering the fragrance/aromatic section occurring earlier in the conclusion.
