# Supplementary material for: Nutritional and physicochemical characteristics of purple sweet corn juice before and after boiling
Source: PLoS One. 2020 May 11;15(5):e0233094. doi: 10.1371/journal.pone.0233094 (PMC7213738; doi:10.1371/journal.pone.0233094)
Supplement: Attachment — Submitted filename: Response to Reviewers 2.docx [file pone.0233094.s004.docx]

Dear Editor,

Thank you and reviewers very much for your valuable comments and constructive advises. We have replied these comments point by point and made revisions in main text.

Reviewer #1: L38: Text: “Several aromatic compounds not previously reported were identified in sweet purple corn …”.

Thanks. It has been revised.

L113: Text: “Purity level of chemicals used for High-Performance Liquid Chromatography (HPLC) and Gas Chromatography Mass Spectroscopy (GCMS) complied with the requirement of the relevant procedures. All other chemicals were of analytical grade”.

Thanks. It has been revised.

L149-150 and L188-189 and L202-203 and L211-212 and L228-229: Text: “The applied process was modified from the national …”.

Thanks. It has been revised.

Comment to earlier remark on statistics in Table 1: Yes, I misunderstood the presentation. You apply both an asterisk the represent statistical significance and essential/semi-essential amino acids. This could be misleading or confusing (evidently). Different symbols could avoid any chance of misinterpretation.

Thanks. A different symbol has been used in Table 1.

Reviewer #2: (No Response)

Reviewer #3: General comment: The authors did a thorough job revising the manuscript and addressed all my questions. I have only a few minor comments and suggestions.

Minor comments:

Line 27: This sentence is awkward. Why would consumer need to know about the physicochemical characteristics of sweet corn juice? Promoting consumer awareness of the nutritional value or improving quality of the product would be more reasonable justifications for the study.

I quite agree with your opinion. In this study, our original purpose was to compare many physicochemical characteristics between raw and boiled sweet corn juices, and by which provide consumers and producers more knowledge about sweet corn juice, and help consumers to make a decision during sweet corn juice consumption. This sentence has been rewritten.

Lines 28-29: “sweet corn juices”.

Thanks. It has been revised.

Line 78: “have been”.

Thanks. It has been revised.

Line 113: “were of analytical grade”.

Thanks. It has been revised.

Line 135: “ANOVA”.

Thanks. It has been revised.

Line 256: “by measuring absorbance at 410 nm using a UV-Vis spectrophotometer”.

Thanks. It has been revised.

Line 267: change “transmissivity” to “transmittance”.

Thanks. It has been revised.
